# GERD assessment including pH metry predicts a high response rate to PPI standard therapy

**DOI:** 10.1186/1471-230X-13-12

**Published:** 2013-01-16

**Authors:** Arne Kandulski, Ulrich Peitz, Klaus Mönkemüller, Helmut Neumann, Jochen Weigt, Peter Malfertheiner

**Affiliations:** 1 Department of Gastroenterology, Hepatology and Infectious Diseases, Otto-von-Guericke University Magdeburg, Leipziger Str. 44, 39120, Magdeburg, Germany; 2 Department of Internal Medicine II and Gastroenterology, Loerstraße 23, 48143, Münster, Germany; 3 Department of Internal Medicine, Gastroenterology, Marienhospital Bottrop, Josef-Albers-Str. 70, 46236, Bottrop, Germany; 4 Department of Medicine 1, University of Erlangen-Nuremberg, Ulmenweg 18, 91054, Erlangen, Germany

**Keywords:** GERD, NERD, PPI, Esomeprazole, Treatment, ph metry, Diagnosis, Therapy

## Abstract

**Background:**

Inadequate response to proton pump inhibitor (PPI) therapy in patients with gastroesophageal reflux disease (GERD) is reported in up to 40%. Patients with non erosive reflux disease (NERD) have lower response rates compared to patients with erosive reflux disease (ERD); pH metry contributes to GERD diagnosis and is critical for proper diagnosis of NERD.

Aim of the study was to assess the need for doubling esomeprazole standard dose (40 mg) for 4 weeks in PPI naive patients with typical reflux symptoms and diagnosis of GERD based on endoscopy and 48 hours, wireless pH metry.

**Methods:**

All patients underwent upper GI endoscopy. Symptoms were recorded with a structured questionnaire (RDQ) and acid exposure was determined by 48 hours, wireless pH monitoring (BRAVO). In case of abnormal acid exposure, patients received a short term treatment with esomeprazole 40 mg q.d. for 4 weeks. If symptoms persisted, patients underwent a second pH metry on PPI and the dose was increased to 40 mg b.i.d.

**Results:**

31 consecutive patients with typical reflux symptoms underwent 48 hours pH monitoring. 22 patients (71%) had abnormal acid exposure, 9 patients had normal pH metry (29%). Of the 9 patients with normal pH metry, 2 were found with erosive esophagitis and 7 without endoscopic abnormalities.

24 patients with documented GERD received esomeprazole treatment. 21 patients achieved complete symptom resolution with 40 mg q.d. after 4 weeks (88%). Only 2 patients required doubling the dose of esomeprazole for complete symptom resolution, 1 patient remained with symptoms.

**Conclusions:**

Patients with typical reflux symptoms and abnormal acid exposure have a high response rate to standard dose esomeprazole regardless of whether they have ERD or NERD.

## Background

GERD is defined as a condition which develops when reflux of gastric contents causes troublesome symptoms and/or mucosal lesions in the distal esophagus [1]. The problems of a symptom-based diagnosis of GERD are demonstrated by Dent and colleagues who found typical symptoms in only 49% of the patients [2] with proven GERD. Nevertheless most guidelines recommend to first administer an empiric trial of proton pump inhibitors (PPIs) for patients presenting with typical GERD-related symptoms without alarm symptoms (dysphagia, weight loss) [3].

Erosive reflux disease (ERD) is diagnosed endoscopically [4,5], however in the absence of erosions, the diagnosis of NERD deserves functional testing. This includes ambulatory pH metry, prolonged pH metry or combined pH and intraluminal impedance measurements to define timing, acid exposure time, reflux characteristics as well as symptom association [3,6,7]. The wireless and prolonged 48 hours capsule pH metry has been demonstrated to exhibit better compliance and patients’ satisfaction and better test accuracy for the diagnosis of GERD due to the prolonged measurement and frequent day-to-day variations in the reflux characteristics of GERD patients [8,9]. Normal acid exposure to the distal esophagus or missing association between reflux episodes and patients’ symptoms are defined as functional heartburn according to ROME III criteria [10].

Adequate acid inhibition with PPI is the current standard therapy for GERD [11,12]. The efficacy in healing reflux esophagitis is very high for PPI with a number needed to treat of 1.7 (95% CI 1.5-2.1) [13]. Furthermore, PPIs are effective for the symptomatic response in GERD [14] but their efficacy differs between the subgroups of ERD and NERD with a larger proportion of non-responders in NERD even when standard dose has been increased to a twice daily dosage [15,16]. We believe that this is most likely due to an incorrect diagnosis of NERD.

Our study was designed to test whether, and in which proportion of patients, PPI standard dose is effective in achieving complete symptom relief if GERD (ERD and NERD) is properly diagnosed by either abnormal endoscopic findings or abnormal acid exposure using 48 hours pH metry. A secondary aim was to determine the proportion of patients that need the escalation of esomeprazole dosage to 40 mg b.i.d for complete symptom relief.

## Methods

The study was approved by the institutional ethics committee at the Otto-von-Guericke University and the German “Bundesinstitut für Arzneimittel und Medizinprodukte” (BfArM), funded by Astra Zeneca, Wedel, Germany (Protocol No. GS0205; Eudract No. 2005-000761-19; Title: Control of Symptoms and Acid Reflux by Esomeprazole in Patients with GERD) and conducted according to the ethical guidelines of the declaration of Helsinki.

### Patients’ population

Patients presenting at the outpatients department of the Department of Gastroenterology, Hepatology and Infectious Diseases with GERD associated symptoms were evaluated. Only patients without prior PPI medication were included in the study (PPI naïve). After given their written informed consent patients were included in the screening (for demographic details see Table 1).

**Table 1:** Demographic data pH data and endoscopic results for patients before therapy with esomeprazole at baseline assessment

	**Screening**	**pH negative**	**symptom relief 40 mg q.d.**	**symptom relief 40 mg b.i.d.**	**persistence**
	**n = 31**	**n = 9**	**n = 19**	**n = 2**	**n = 1**
**Gender (male/female)**	12/19	0/9	9/10	2/0	1/0
**Age (mean±SD)**	52.4±17.1 years	47.5±3.5 years	52.5± 2.8 years	23.7± 5.1 years	66 years
**Endoscopy**					
NERD		7 (no erosions)	7		
ERD Los Angeles A		2	4	1	1
ERD Los Angeles B			5	1	
ERD Los Angeles C			1		
Barrett’s Esophagus			2		
**DeMeester score ±SD**	_	5.5 ± 1.8	26.9 ± 3.9	20.9 ± 15.7	30.3
‐ DeMeester [day 1]	_	5.9 ± 2.9	28.2 ± 4.8	21.6 ± 19.8	23.2
‐ DeMeester [day 2]	_	4.9 ± 2.4	25.6 ± 3.9	10.7 ± 3.5	34.8

### Objectives and study design

The primary objective was to determine the proportion of patients that achieve complete symptom relief with esomeprazole 40 mg q.d. or b.i.d. Complete symptom relief was defined as absence of reflux symptoms during seven days, as assessed by the self-administered Reflux Disease Questionnaire (RDQ) and a diary. Secondary, the response rates for symptomatic response for proper diagnosed NERD were assessed and related to ERD.

A further objective was to assess the relation between gastrointestinal symptom pattern and 48 hour acid reflux profile during esomeprazole treatment in patients with incomplete symptom relief.

The study was designed as an open, mono-centric treatment study with measurement of symptoms and pH monitoring before and during therapy with esomeprazole. The diagnosis of GERD was confirmed by 48 hours BRAVO pH monitoring and/or erosions during upper GI endoscopy.

In case of abnormal findings in BRAVO pH monitoring, patients entered a short term treatment (I) with esomeprazole 40 mg q.d. for 4 weeks. During acid suppressive therapy, symptom severity was again assessed by RDQ and a symptom diary. Complete symptom relief was defined as no GERD symptoms during the last 7 days as documented in the diary and in the RDQ questionnaire.

**Figure 1:**
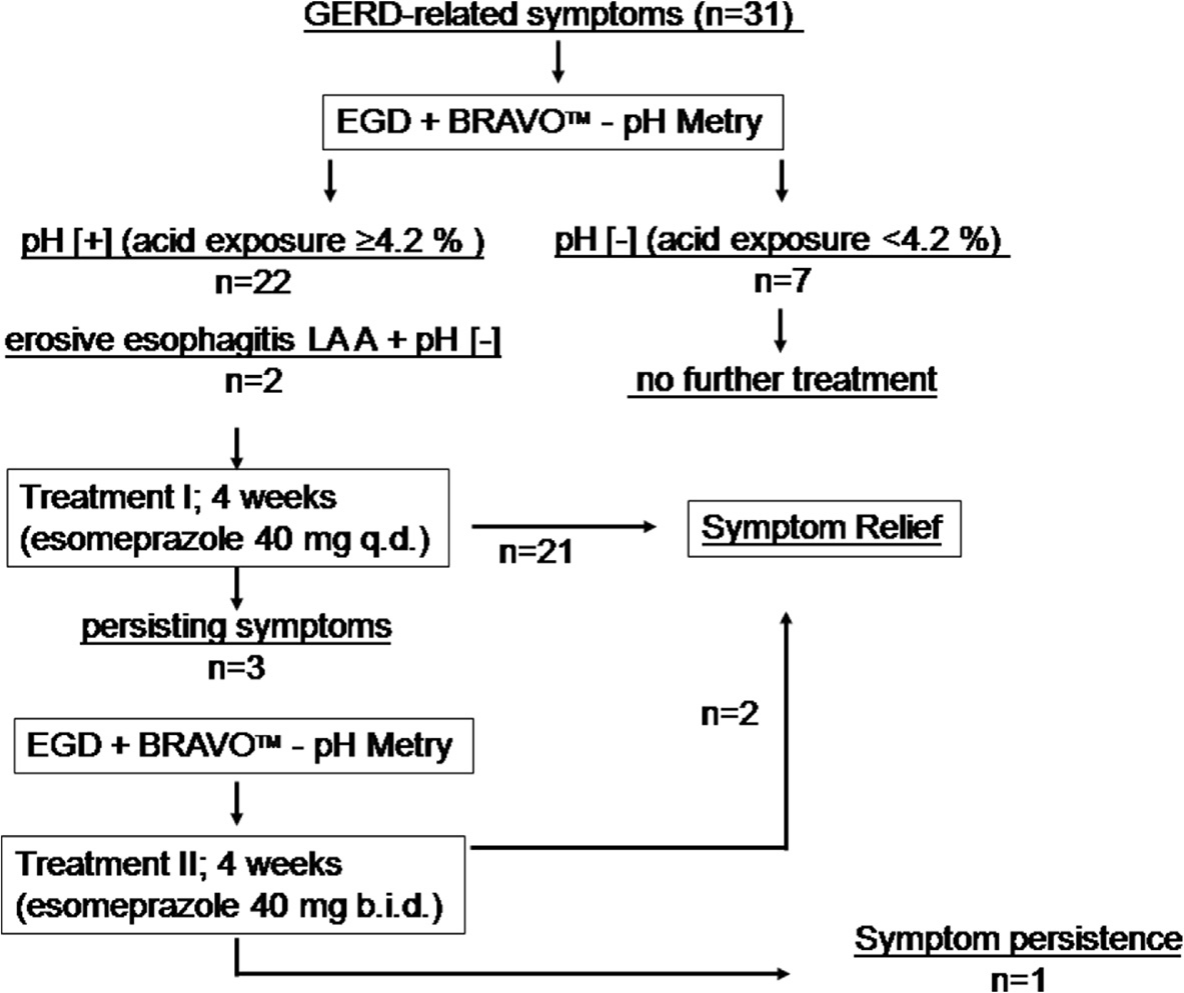
Study design and disposition of patients.

In case of persisting symptoms, the patients underwent a second diagnostic EGD and functional testing, followed by escalating dosage (II) with esomeprazole 40 mg b.i.d. for another 4 weeks. The symptom relief was evaluated under escalating dosage as described before (Figure 1).

The study medication was to be ingested 30 minutes before breakfast (I) and dinner (I, II) with 100 ml of table water.

### Esophagogastroduodenoscopy (EGD)

After an overnight fast, all patients underwent EGD under intravenous conscious sedation (midazolam 2–5 mg) using a standard videogastroscope (GIF Q160, Olympus Optical Europe, Hamburg, Germany). Endoscopic esophageal landmarks were defined as the gastroesophageal junction with the beginning of the gastric folds and the Z-line as the squamocolumnar junction. Erosive esophagitis was characterized according to Los Angeles classification [4].

NERD was defined as normal appearing GEJ and abnormal acid exposure during 48 hours pH metry.

### Wireless 48 hours BRAVO™ pH monitoring

Ambulatory pH monitoring was performed over 48 hours using the wireless BRAVO capsule pH monitoring device (Medtronic, Minneapolis/GIVEN). Capsules were calibrated at pH 1.0 and 7.0 by submersion in buffer solutions (Medtronic/GIVEN) according to the product information. During EGD the gastroesophageal junction (GEJ) was visualized and the capsule was attached at the esophageal mucosa at 6 cm above GEJ with vacuum suction of 700 mmHg for 2 minutes. The correct placement of the capsule was confirmed endoscopically. The pH data was transmitted by the capsule to a recording device with 433 Hz and a sampling interval of 6 seconds. The patients were asked to carry or keep the recording device within a maximum distance of 100 cm maximum from their bodies. The patients were instructed to follow their normal daily activities and diet. During the period of 48 hours, meal time, sleep disturbances, supine and upright positions were marked in a patients’ diary.

After 48 hours patients returned to hospital and the data were downloaded from the recording device. The recordings were completed by entering the diary information manually and analyzed based on the manufacture’s software (POLYGRAM NET™ Version 14.1.1322.287).

Total numbers of acid episodes, acid exposure time (AET, pH < 4) and DeMeester score were analyzed for day 1 and day 2 separately as well as for 48 hours in total. Acid exposure time ≥ 4.2% and/or a DeMeester score ≥ 13.9 were considered abnormal [6].

### Evaluation of symptoms by validated reflux disease questionnaire (RDQ) and patient’s diary

Complete symptom relief was defined as absence of GERD-related symptoms during the last 7 days as documented by RDQ (<5 points) and a symptom-assessing diary. The self-administered patient’s diary documented the severity of symptoms on 7 days before and under treatment with esomeprazole. The diary graded heartburn objectively on a 5 point Likert-scale for each day as used in the EXPO study [17].

The RDQ was designed to grade different reflux symptoms during the last seven days. The RDQ is a self-administered questionnaire in which subjects are asked to report the frequency and severity of their upper gastrointestinal symptoms. There are three subscales that evaluate regurgitation, heartburn, and dyspepsia. Response options were also scaled as Likert-type with scores ranging from 0 to 5 for frequency and severity. Each subject’s score was calculated as the mean of item responses with higher scores indicating more severe or frequent symptoms [18,19].

### Statistical analysis

According to the results of the EXPO study, where 91.1% of patients experienced had complete symptom relief after four week treatment with esomeprazole 40 mg orally the sample size was calculated. With focus on complete symptom relief, the hypothesis H0 complete symptom relief < =68% vs. H1 complete symptom relief > 85% was tested by a one-sided binomial test, which will have a power of 80% (type-I-error 5%). 40 patients were calculated as the requested sample size.

All data entered into a database using the Microcal Origin™ 5.0 program package (Northhampton, MA, USA) and SPSS^©^ 12.0. Data is expressed as mean and 95%-CI (confidence intervals), if not stated otherwise. For statistical analysis (pre- and post-treatment) parametric *T*-test was used. All test were applied two-sided with a level of significance of P < 0.05.

## Results

### Patients’ characteristics

A total of 40 patients with predominantly female gender were included in the screening phase of the study (mean age 52 years; range: 18–79 years) (Table 1).

31 patients met the inclusion criteria with either endoscopic findings of erosive reflux disease (n = 2), abnormal pH metry (NERD n = 7) or both (n = 15). In 7 patients with normal appearing gastroesophageal junction (all female), GERD was excluded by normal results in BRAVO pH monitoring (Table 2).

**Table 2:** Diagnosis and response to esomperazole 40 mg q.d. for 4 weeks

**diagnosis**	**n**	**response to esomeprazole 40 mg q.d.**
abnormal pH metry only (NERD)	7	100%
ERD and abnormal pH metry [pH +]	15	80%
ERD and normal pH metry [pH -]	2	100%
normal endoscopy and normal ph metry [pH-]	7	no therapy

There was a withdrawal of 9 patients for different reasons: 5 patients did not complete neither questionnaire nor diary; one patient stopped the study medication because of an newly appeared exanthema; 3 patients presented with technical problems in BRAVO capsule testing (1 patient suffered from severe chest pain that required endoscopic removal of the capsule; 2 patients documented an early drop off the capsule).

### Control of symptoms and acid reflux by standard dose and doubled standard dose of esomeprazole

Finally, 24 patients entered the treatment phase. Endoscopic diagnosis revealed 7 patients with non-erosive reflux disease (NERD), 15 patients with erosive reflux disease (ERD) and 2 patients with newly diagnosed short segment Barrett’s esophagus without dysplasia (Tables 1, 2).

After 4 weeks of treatment with esomeprazole 40 mg q.d., 21 (88%) patients achieved complete symptom relief. 2 patients achieved symptom relief after escalating dosage of esomeprazole to 40 mg b.i.d. (8%), but only 1 patient presented with persisting symptoms even after escalating dosage. In this patient, pH metry revealed an even unchanged cluster of pH metry during esomeprazole treatment (Figure 1).

**Figure 2:**
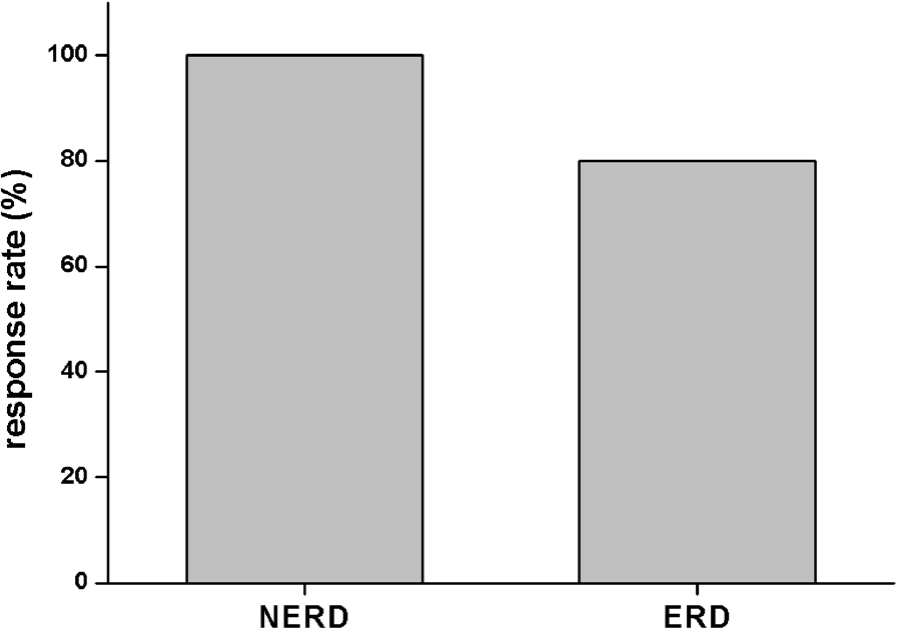
Therapeutic response to esomeprazole 40 mg o.d. in patients with NERD and ERD.

No differences were obtained between patients with NERD and ERD (Figure 2) and no substantial differences were found between day 1 and day 2 of pH analysis (Table 1).

### Relief of symptoms documented RDQ and patient’s diary

**Figure 3:**
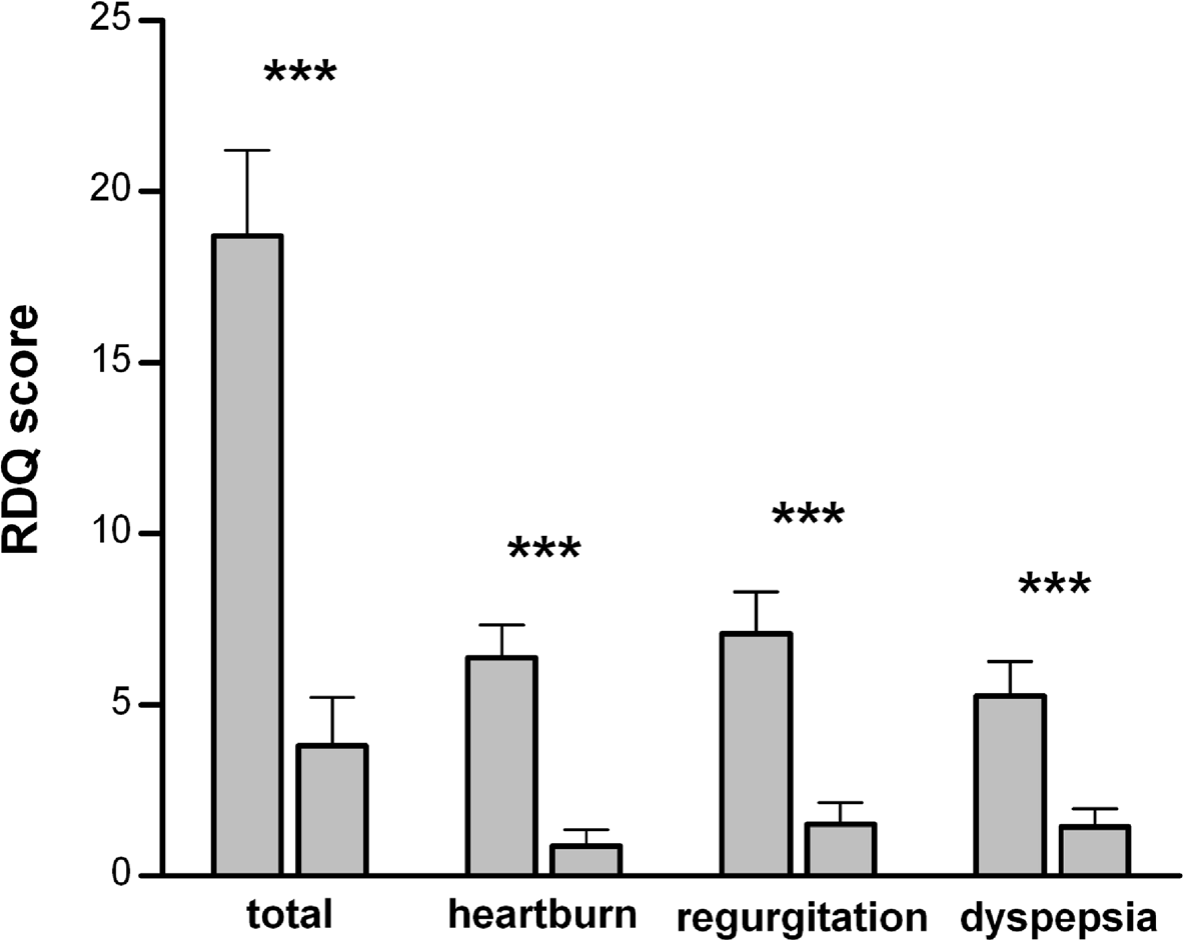
Mean heartburn, regurgitation, dyspepsia and total RDQ scores at screening and after 4 weeks of treatment.

**Figure 4:**
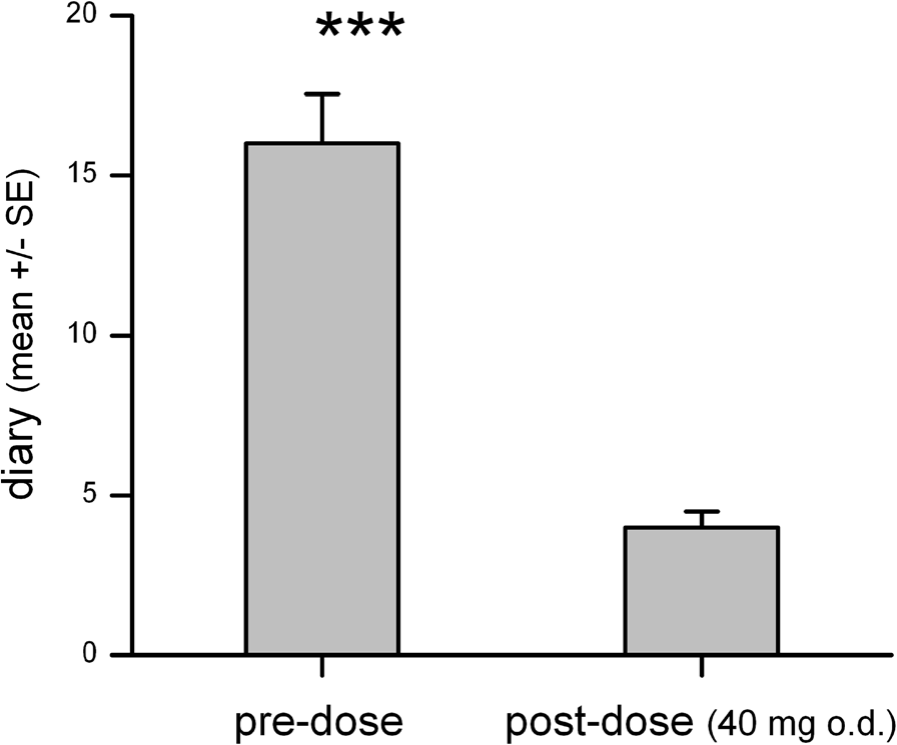
Mean severity of symptoms at screening and after 4 weeks of treatment with esomeprazole 40 mg q.d. according to patients’ diary.

Patients with complete relief of symptoms according to RDQ and diary are shown in Figure 3. The calculated RDQ means pre- and post PPI therapy differed 6.3–times in total (19.1 [14.07 – 24.02] vs. 3.2 [0.51 – 5.4]; p < 0.0001), 8.2-times for heartburn (6.5 [4.5 – 8.4] vs. 0.8 [0.19 – 1.78]; p < 0.0001), 5.1-times for regurgitation (7.2 [4.7 – 9.63] vs. 1.4 [0.08 – 2.75]; p < 0.0001) and 4.2-times for dyspepsia (5.4 [3.36 – 7.47] vs. 1.3 [0.17 – 2.3]; p < 0.0001). Similar to the RDQ, the diaries were evaluated for the patients included and documented a 3.9-fold reduction of the mean value (17.2 [13.9 – 20.49] vs. 4.4 [3.3 – 5.1]; p < 0.0001; Figure 4).

## Discussion

The main finding of this study is that patients with typical reflux symptoms and abnormal acid exposure have a high response rate to standard esomeprazole regardless of whether they have ERD or NERD. Two thirds (22/31) of patients with typical GERD-related symptoms had an abnormal acid exposure in esophageal BRAVO pH metry. Including 2 patients with erosive changes but normal pH metry, 24 patients were eligible for PPI treatment in our study.

88% of this well selected patient group achieved complete symptom relief on esomeprazole standard dose for 4 weeks. Symptomatic response was similarly obtained in patients with ERD and NERD (Table 2; Figure 2). The claim that patients with NERD would have a worse response to PPI is therefore most likely due to the inclusion of patients without abnormal gastroesophageal reflux in previous studies. Misdiagnosis of GERD – NERD in particular – might also explain reasonably the high PPI failure rate in previously published data. Weijenborg and colleagues systematically reviewed previous outcome studies and found only 2 studies defining NERD by both negative endoscopy and a positive pH-test. In contrast to poor response rates in empirical treated or endoscopy-negative patients, the pooled estimate rate of complete relief of heartburn after 4 weeks of for those accurately diagnosed NERD was 0.73 (95% CI 0.69-0.77) and comparable to patients with ERD [20]. This clinical data indicates to careful asses the diagnosis of NERD and differentiate especially from functional heartburn to predict a therapeutic success of current PPI therapy.

We excluded patients with normal acid exposure as there is no rationale for PPI treatment. This category of patients is likely to account for the frequent reports with up to 30-40% PPI failure to standard dose [15,21]. In routine practice in Germany, the response rate to PPI is 60% [22]. For patients not responding to PPI in presence of typical symptoms, functional testing is performed to test the initial diagnosis and to further investigate for conditions that might explain PPI refractoriness. Among them, persistent acid or non-acid reflux episodes have been reported to be responsible for incomplete symptom relief [6,23-26].

In a further subset of patients, reflux symptoms may be unrelated to reflux episodes at all and related to a functional syndrome (functional heartburn) [27]. Although unable to determine the proportion of “non acidic” reflux episodes by BRAVO pH metry, our study reemphasizes the importance of the patients’ interview and interpretation of symptoms to distinguish between acid-related symptoms and functional disorders that often overlap and requires different medical treatment [27,28].

For patients not responding to PPI, pH metry should be considered to confirm the diagnosis of abnormal gastroesophageal reflux. Mechanisms involved in symptom generation or perpetration are either hypersensitivity to visceral stimuli or weakly acidic reflux episodes, a fast hepatic metabolism of PPIs [29] or duodenogastroesophageal reflux (DGER) [15,30]. Intestinal proteases in the refluxate and interaction with epithelial protease-activated receptors are also involved in the pathogenesis of mucosal inflammation in GERD pathogenesis [31,32].

The shortcomings of the study are the missing control group and the small sample size. This was mainly due to the inclusion criteria of PPI naive patients in a referral centre. As calculated before, the recruitment was finalized after having screened 40 patients.

In spite of the small sample size, the results indicate daily clinical practice. Nevertheless, our study has the true advantage of having included truly PPI-naive patients, a fact that is very hard in routine clinical practice, as most physicians administer PPI very quickly based on current guidelines. However, this “aggressive” approach might need to be rethought, as we believe that many patients receiving PPI do not suffer from NERD or ERD, and thus being over treated. Thus, a careful initial assessment of symptoms combined with functional testing may identify the patients who respond well to PPI therapy. This fact needs to be reconsidered in the interpretation of many clinical trials concerning response to PPI therapy, especially in NERD [20].

## Conclusion

PPI naïve patients with characteristic GERD-related symptoms and abnormal findings in pH metry had an excellent response to standard dose esomeprazole. Due to the small sample size of our study it cannot be concluded, but patients with NERD diagnosed with pH metry and endoscopy did not differ in their response rates to esomeprazole in comparison with ERD. This corresponds to the systematic review cited above and responds to the studies investigating the PPI test, and documented symptom relief in up to 90% in case of pathological acid exposure [33,34].

29% of the patients in our study suffered from typical GERD-related symptoms but had no abnormal acid exposure in 48 hours pH metry, predominantly with unsuspicious results in EGD (no erosions). This may partly explain the high proportion of PPI non-responsiveness in the literature, since the patients may all have been grouped as NERD [21].

For non-responders with abnormal 48 hours pH metry, in clinical practice it may be appropriate to escalate PPI to double standard dose before embarking in functional testing (MII-pH) and seek for other mechanisms in GERD pathogenesis.

## Competing interests

The authors declare that they have no competing interests.

## Authors’ contributions

PM, UP and KM designed the study. AK, HN and JW enrolled the majority of patients. AK and JW provided pH metry analyses and clinical data. The manuscript was drafted by AK, UP and KM and reviewed for important intellectual content by JW, HN and PM. All authors read and approved the final manuscript.

## Pre-publication history

The pre-publication history for this paper can be accessed here:

http://www.biomedcentral.com/1471-230X/13/12/prepub
